# Supplementary material for: Glucose Limitation Sensitizes Cancer Cells to Selenite-Induced Cytotoxicity via SLC7A11-Mediated Redox Collapse
Source: Cancers (Basel). 2022 Jan 11;14(2):345. doi: 10.3390/cancers14020345 (PMC8773648; doi:10.3390/cancers14020345)
Supplement: Supplementary file 1 [file cancers-14-00345-s001.zip › cancers-1505706-supplementary.pdf]

Supplementary Material

Glucose Limitation Sensitizes Cancer Cells to Selenite-Induced Cytotoxicity via SLC7A11-Mediated Redox Collapse

Hui Chen, Han Zhang, Lixing Cao, Jinling Cui, Xuan Ma, Chong Zhao, Shutao Yin and Hongbo Hu

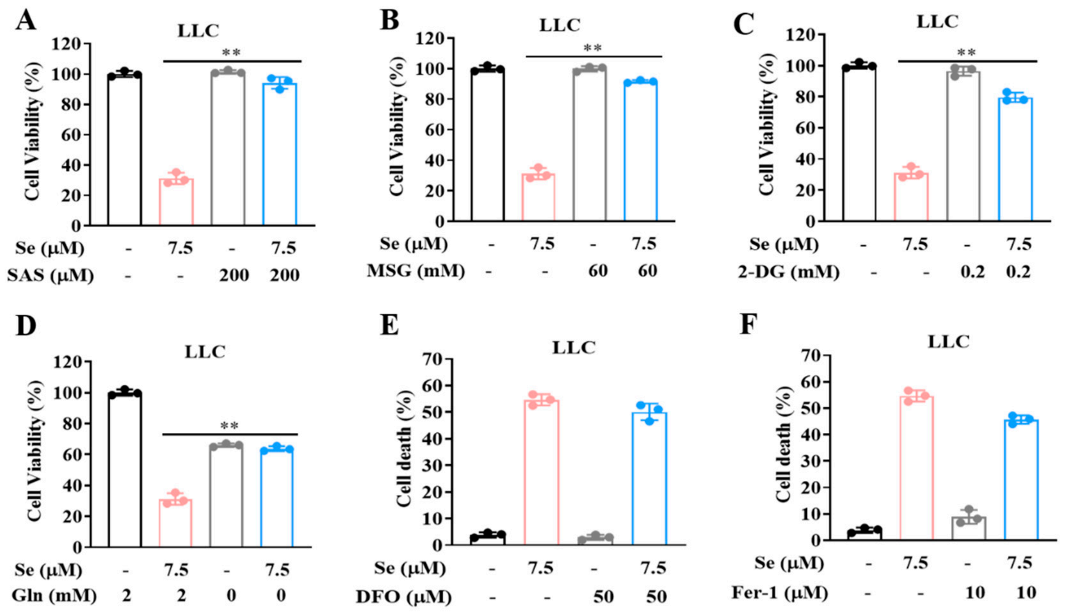

Figure S1. SLC7A11 inhibitors or 2-DG protect LLC cells from selenite/glucose deprivation-induced cytotoxicity, and selenite/glucose deprivation-induced cytotoxicity to LLC cells is independent of ferroptosis. (A-C) Cell viability of LLC cells cultured in the medium containing 2.5 mM glucose with or without treatment of selenite or SAS/MSG/2-DG for 24 h. (D) Cell viability of LLC cells cultured in the medium containing 2.5 mM glucose with or without treatment of selenite or glutamine deprivation for 24 h. (E, F) Cell death of HCT116 cells cultured in the medium containing 2.5 mM glucose with or without treatment of selenite or DFO or Fer-1 for 24 h. Results are representative of three biologically independent experiments. Data are expressed as mean±SD, ** $p<0.01$. Se: selenite; SAS: salicylazosulfapyridine; MSG: monosodium glutamate; 2-DG: 2-deoxy-D-glucose; Gln: glutamine; DFO: deferoxamine mesylate; Fer-1: ferrostatin-1.

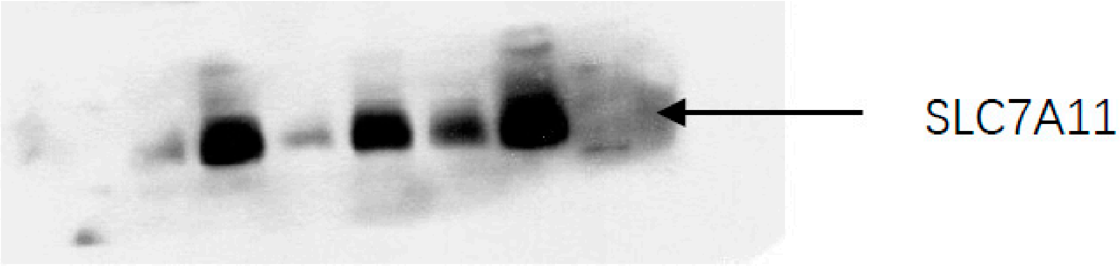

Fig.3A HCT116-SLC7A11

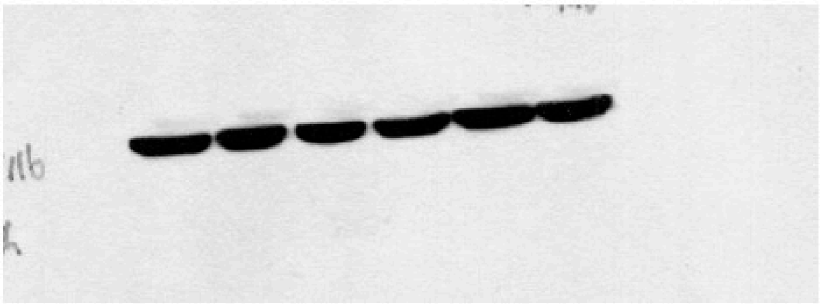

Fig.3A HCT116-β-actin

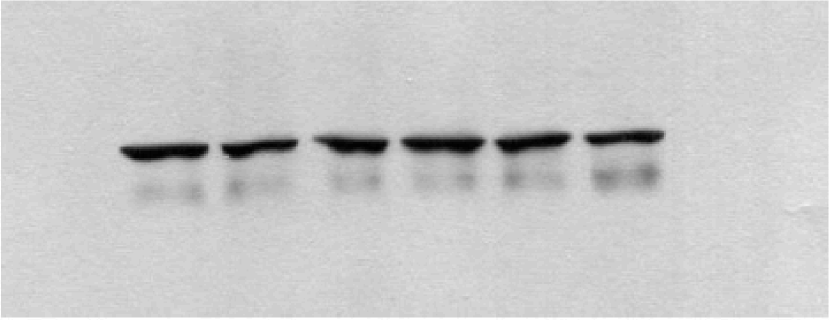

Fig.3F A549-β-actin

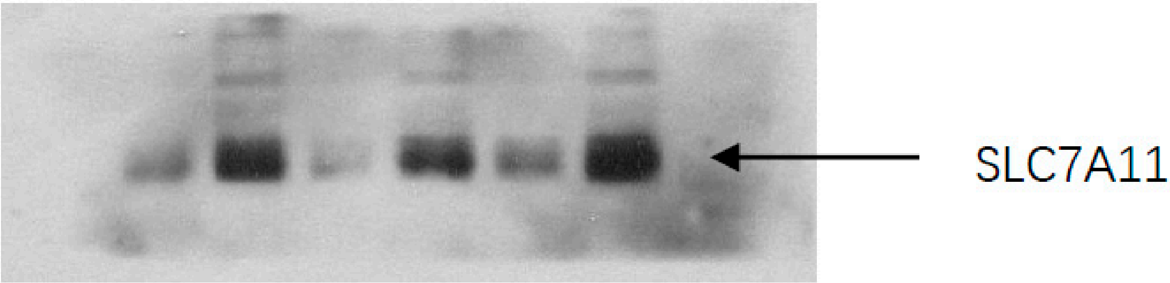

Fig.3F HePG2-SLC7A11

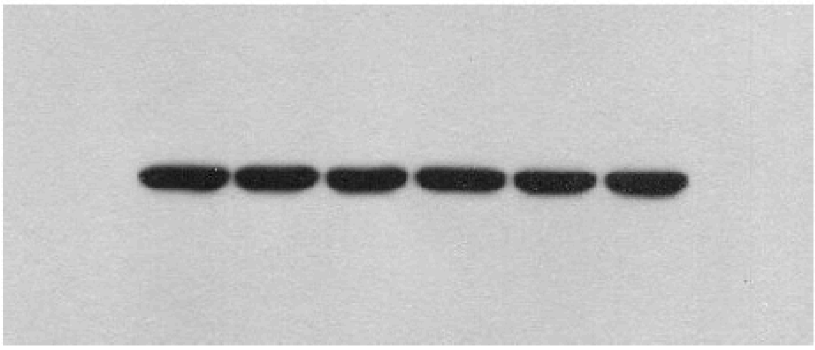

Fig.3F HePG2-β-actin

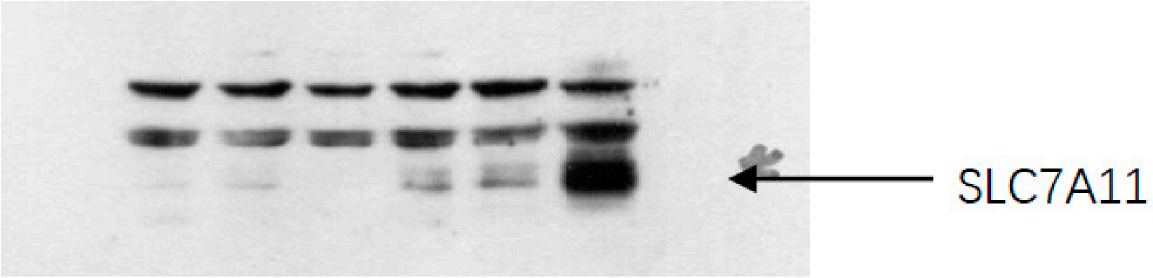

Fig.3F LLC-SLC7A11

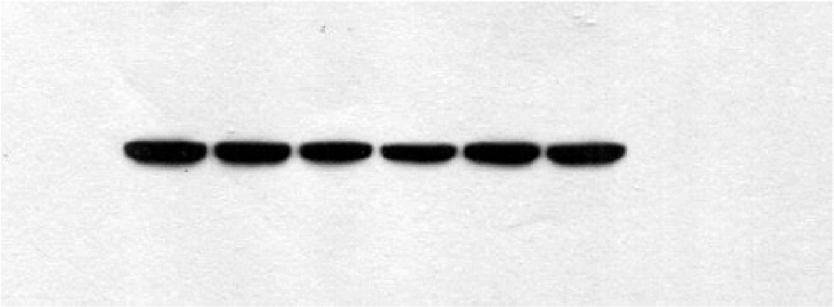

Fig.3F LLC-β-actin

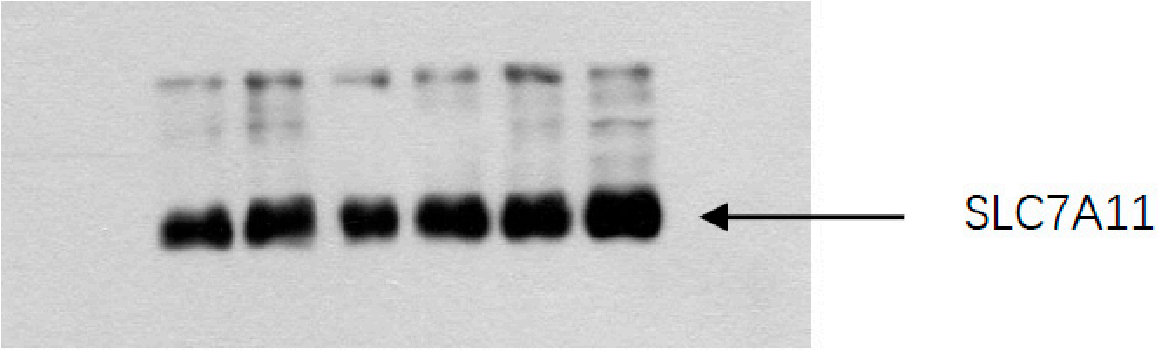

Fig.3F A549-SLC7A11

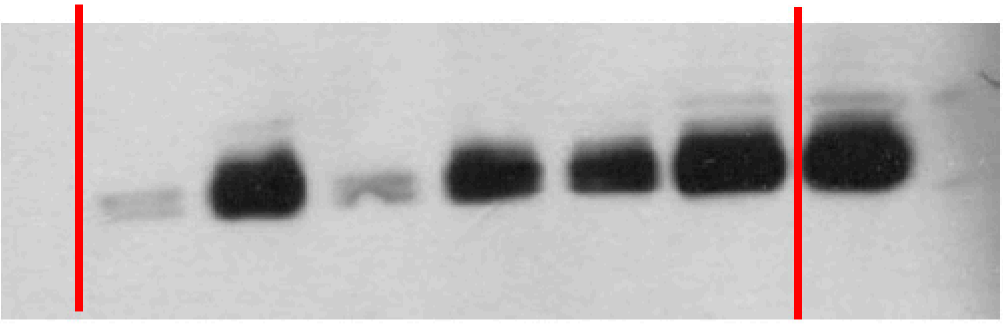

Fig.3F MDA-MB-231-SLC7A11

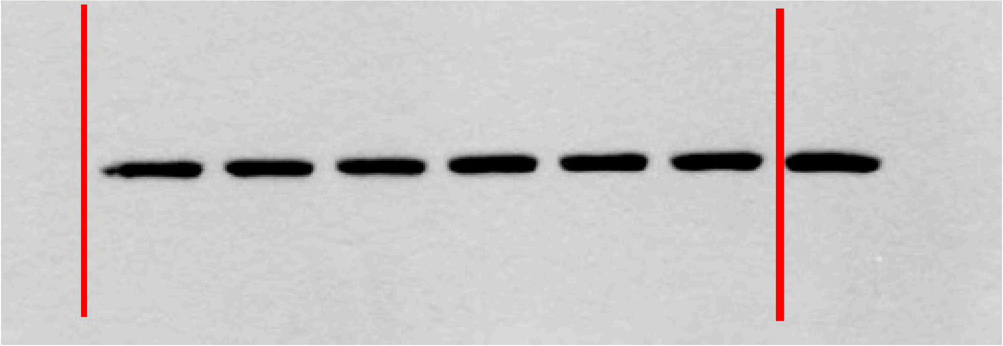

Fig.3F MDA-MB-231-β-actin

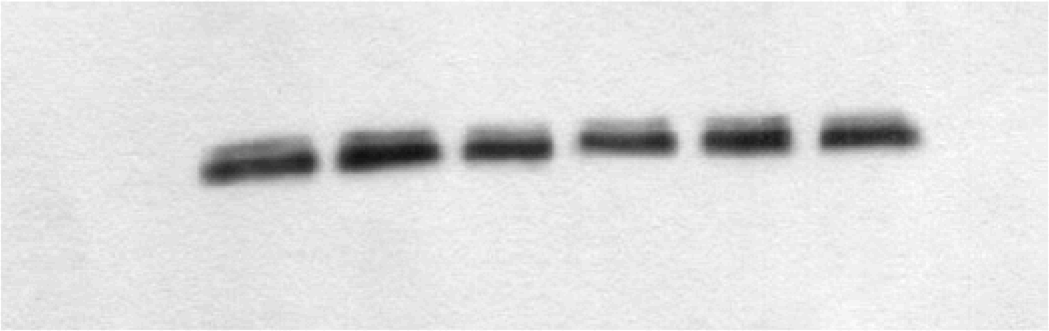

Fig.3F HK2-SLC7A11

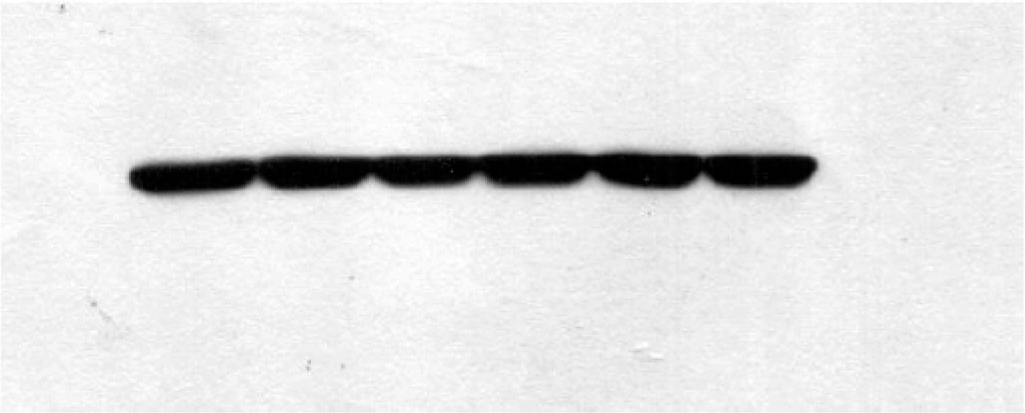

Fig.3F HK2-β-actin

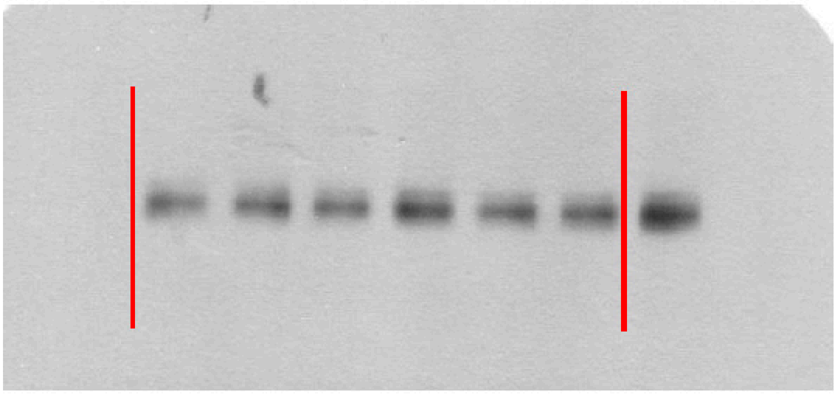

Fig.3F HCT116-SLC7A11

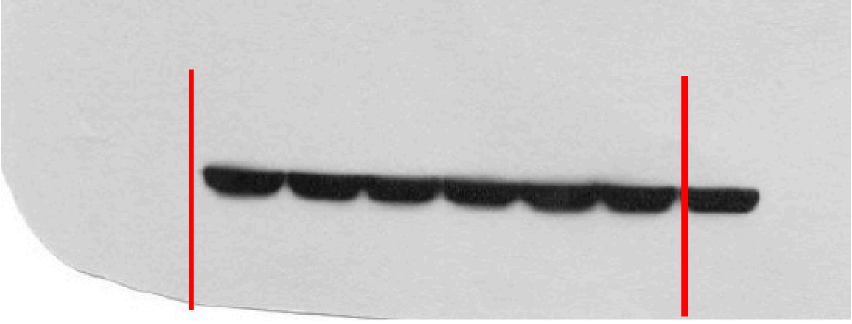

Fig.3F HCT116-β-actin

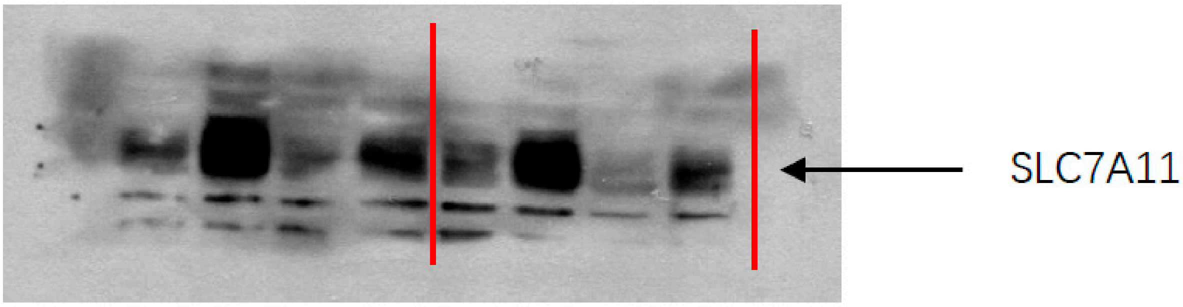

Fig.4I HCT116-SLC7A11

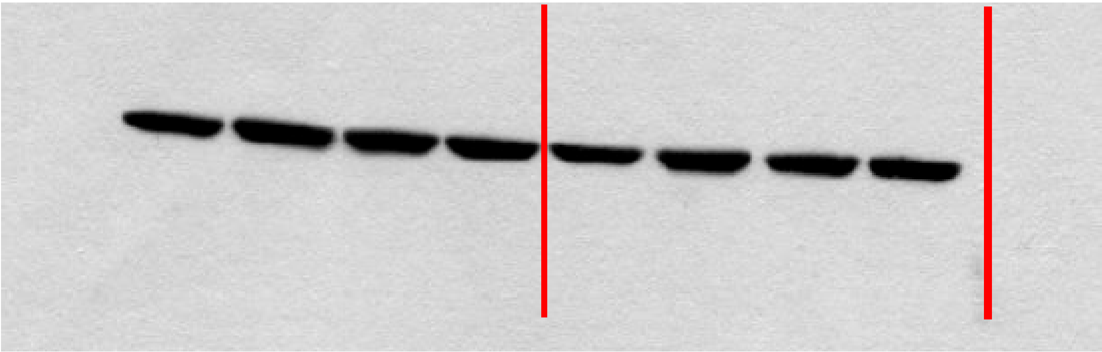

Fig.4I HCT116-β-actin

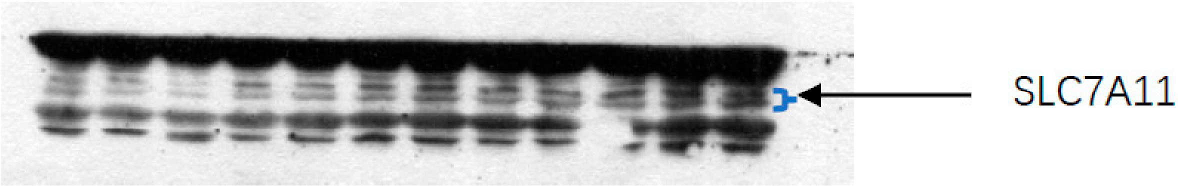

Fig.6E HCT116 tumor-SLC7A11

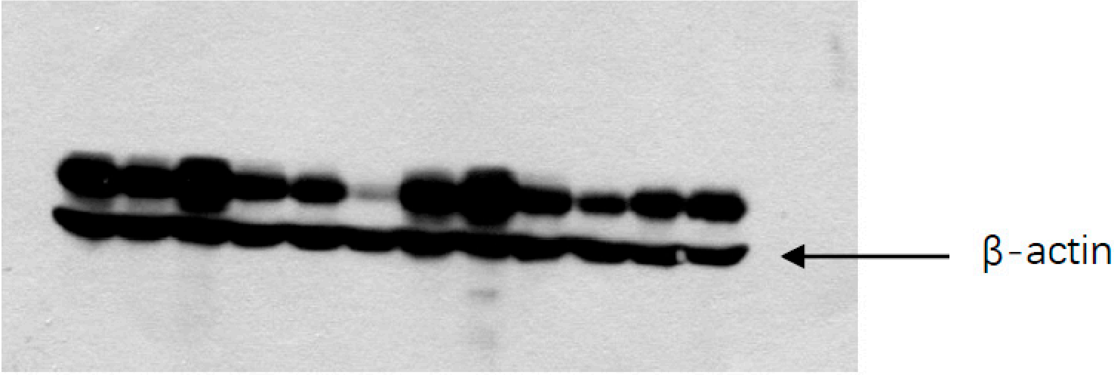

Fig.6E HCT116 tumor-β-actin

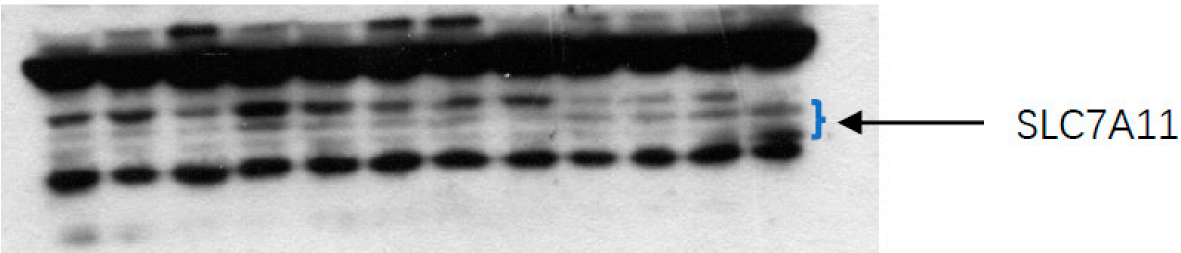

Fig.6E HCT116 tumor-SLC7A11

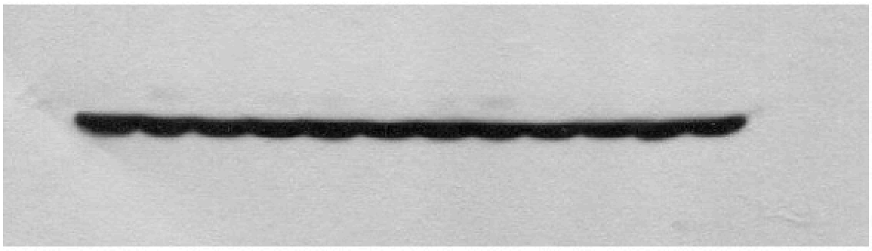

Fig.6E LLC tumor-β-actin

Figure S2. Full western bolts figure.
